# Management of a large percutaneous endoscopic gastrostomy tube-associated gastric ulcer with endoscopic suturing

**DOI:** 10.1055/a-2155-4941

**Published:** 2023-08-30

**Authors:** Benjamin Norton, Nasar Aslam, Apostolis Papaefthymiou, Andrea Telese, Charles Murray, Rehan Haidry

**Affiliations:** 1 Digestive Diseases and Surgery Institute, Cleveland Clinic London, London, UK; 2 Department of Gastroenterology, University College London Hospitals NHS Foundation Trust, London, UK

Insertion of a percutaneous endoscopic gastrostomy (PEG) tube is a common procedure performed predominantly for provision of enteral nutrition. Serious complications requiring intervention are reported in 0.4 %–4.4 % of cases 1 . Among these, PEG-associated gastric ulcers are a rare complication, thought to be secondary to pressure necrosis, which can predispose to upper gastrointestinal bleeding 2 . Like other gastric ulcers, these can be treated with conventional endoscopic therapy; however, when treatment is unamenable or fails, endoscopic suturing provides a novel management option that has been described in a few case reports with good results 3 4 .

A 47-year-old woman receiving longstanding enteral feeding through a PEG with jejunal extension presented with anemia (hemoglobin 89 g/L) and suspected upper gastrointestinal bleeding. Gastroscopy showed a 3-cm PEG-associated ulcer ( Fig. 1 ) with a visible vessel. The PEG was removed, but conventional endoscopic therapy was unsuccessful. She subsequently underwent vessel cauterization with a heater probe under red dichromatic imaging (RDI), followed by endoscopic suturing using the Apollo OverStitch Sx ( Video 1 ). At follow-up endoscopy 6 weeks later, there was evidence of successful ulcer healing, with the sutures still in situ ( Fig. 2 ).

**Fig. 1:**
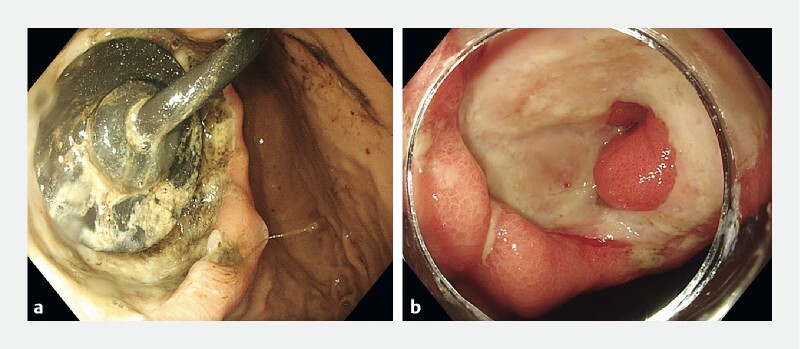
Endoscopic images showing a percutaneous endoscopic gastrostomy (PEG) tube-associated gastric ulcer: **a** with the PEG in situ; **b** after removal of the PEG.

**Video 1** A percutaneous endoscopic gastrostomy (PEG)-associated ulcer on the anterior stomach wall is treated with a heater probe to the vessel using red dichromatic imaging (RDI), then by endoscopic suturing with a continuous running suture followed by a cross suture.


**Fig. 2:**
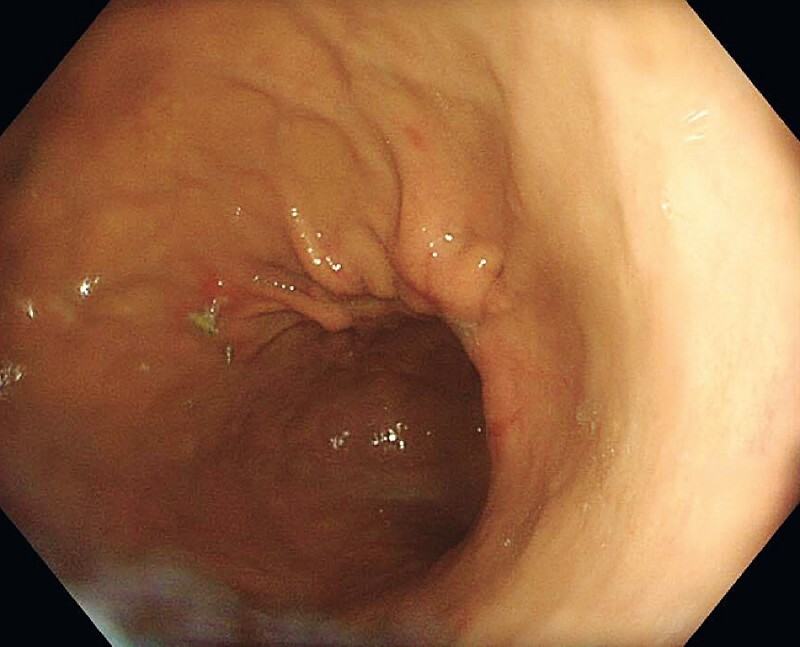
Endoscopic image 6 weeks after endoscopic suturing showing complete gastric ulcer healing, with evidence of residual sutures present.

Endoscopic suturing using the Apollo OverStitch Sx is a safe and effective technique for the management of gastric ulcers that are not amenable to or fail conventional endoscopic therapy.

Endoscopy_UCTN_Code_TTT_1AO_2AI

## Notes

Endoscopy E-Videos

https://eref.thieme.de/e-videos
*E-Videos* is an open access online section of the journal *Endoscopy* , reporting on interesting cases and new techniques in gastroenterological endoscopy. All papers include a high-quality video and are published with a Creative Commons CC-BY license. Endoscopy E-Videos qualify for HINARI discounts and waivers and eligibility is automatically checked during the submission process. We grant 100% waivers to articles whose corresponding authors are based in Group A countries and 50% waivers to those who are based in Group B countries as classified by Research4Life (see: https://www.research4life.org/access/eligibility/ ). This section has its own submission website at https://mc.manuscriptcentral.com/e-videos
